# Fostering affect-related competencies and positive affective exercise experiences for promoting a physically active lifestyle in inactive young adults: study protocol for the FEEL cluster randomized controlled trial

**DOI:** 10.1186/s12889-025-24374-9

**Published:** 2025-11-28

**Authors:** Martin Bührer, Stephanie Rosenstiel, Hannah Besel, Daniel Leyhr, Gorden Sudeck, Julia Schmid

**Affiliations:** 1 Institute of Sport Science, University of Bern, Bremgartenstrasse 145, Bern, CH 3012 Switzerland; 2 Institute of Sports Science & Methods Center, Eberhard Karls University of Tübingen, Tübingen, Germany; 3 Interfaculty Research Institute for Sport and Physical Activity, Eberhard Karls University of Tübingen, Tübingen, Germany

**Keywords:** Pleasure, Motivation, Body-inclusive spaces, Experiential learning, Health literacy, Physical literacy, Exercise Adherence, Well-being

## Abstract

**Background:**

Growing evidence highlights the role of affective responses in shaping exercise behavior. Various factors, such as social setting, intensity, or activity type, influence affective exercise experiences. However, previous interventions have typically targeted only one factor at a time. The idea of the FEEL exercise program is to address these various factors in a holistic manner. Additionally, it focuses on fostering Physical Activity-Related Affect Regulation (PAAR) competence in participants, which is crucial for maintaining regular exercise behavior and well-being.

**Methods:**

The FEEL study is a multicenter, cluster-randomized controlled trial. It evaluates the FEEL exercise program's efficacy compared to an active control group participating in a standardized Functional Training program. A total of 160 young adults (aged 18–35) who are not regularly exercising will be recruited across the two cities, Tübingen (Germany) and Bern (Switzerland). Both exercise programs consist of eight weekly 90-min group-based sessions. Primary outcome will assess PAAR using a validated scale, while secondary outcomes will include affective exercise experiences (AFFEXX), motivational competence, physical activity levels, and affective well-being. Measurements will be collected at baseline (t1), post-intervention (t2), and at an 8-week follow-up (t3). Data will be analyzed using multilevel modeling to examine group-by-time interactions, following a modified intention-to-treat approach.

**Conclusions:**

This study explores the impact of a holistic intervention in a primary and secondary prevention context. It may provide valuable insights for practical considerations of affective processes and related competencies in exercise programs.

**Trial registration (retrospectively on the 31.10.2024):**

German Clinical Trials Register; ID: DRKS00035302.

**Supplementary Information:**

The online version contains supplementary material available at 10.1186/s12889-025-24374-9.

## Background

Physical inactivity remains a major public health challenge in the twenty-first century [1]. Although numerous individual-level programs have been implemented to promote physical activity (PA), their overall impact has been limited [2]. One potential reason for this modest effectiveness is the predominant reliance on (social-) cognitive frameworks (e.g., theory of planned behavior or social cognitive theory; [3]).

However, recent scientific evidence suggests that emphasizing affective processes may enhance the effectiveness of interventions targeting PA [4–7]. According to the Affective-Reflective Theory (ART; [8]), repeated affective experiences associated with exercise gradually shape a positive or negative “affective valuation” towards it. Consequently, when an individual encounters an exercise-related stimulus, this valuation automatically activates the corresponding affective state (e.g., displeasure) along with an associated action impulse (e.g., avoidance). Furthermore, because affective processes are continuously salient, they precede and influence subsequent cognitive or reflective processes during exercise [8].

To better describe affective valuation towards exercise built over the life course of an individual Ekkekakis et al. [9] introduced the Affective Exercise Experiences (AFFEXX) framework. This framework outlines the core affective exercise experiences, summarizing valanced designations towards exercise across the three dimensions: Pleasure-Displeasure, Energy-Tiredness, and Calmness-Tension [10]. Core affective exercise experiences are influenced by six antecedent appraisals. Examples include Empowerment vs. Damage from exercise, Pride/Honor vs. Shame/Guilt about one’s exercise behavior, and Interest vs. Boredom in exercise. Together with the core affective experiences, these appraisals shape an individual’s attraction to or aversion toward exercise. Research has shown that the AFFEXX constructs predict exercise behavior more effectively than traditional factors such as intention or self-efficacy [9].

The foregoing considerations highlight the importance of designing interventions that provide individuals with pleasant exercise experiences, as such experiences significantly increase the likelihood of maintaining regular exercise [11, 12]. To achieve sustainable behavioral change, however, it is equally essential to empower individuals to engage in exercise autonomously in ways that elicit positive affective responses. Empirical evidence suggests that inactive individuals, in particular, lack this so-called competence in physical activity-related affect regulation (PAAR) [13–17]. Moreover, emerging empirical evidence suggests that PAAR may moderate the relationship between physical activity and affective well-being [17–19]. Addressing this requires not only fostering an understanding of the factors influencing affective responses to exercise (e.g., intensity, type of activity) but also enabling individuals to apply this knowledge effectively in their own practice [13, 20]. Existing intervention studies typically focus on single factors influencing affective responses to exercise, such as exercise intensity (e.g., [21]), music (e.g., [22]), green environment (e.g., [23]), or activity type (e.g., [24]). While these studies provide valuable insights, they fail to account for the complex interplay of multiple factors that collectively shape affective responses [25] and how to enable inactive people to regulate their affect on their own. Therefore, there is a critical need for holistic programs that simultaneously address multiple factors.

**Fig. 1:**
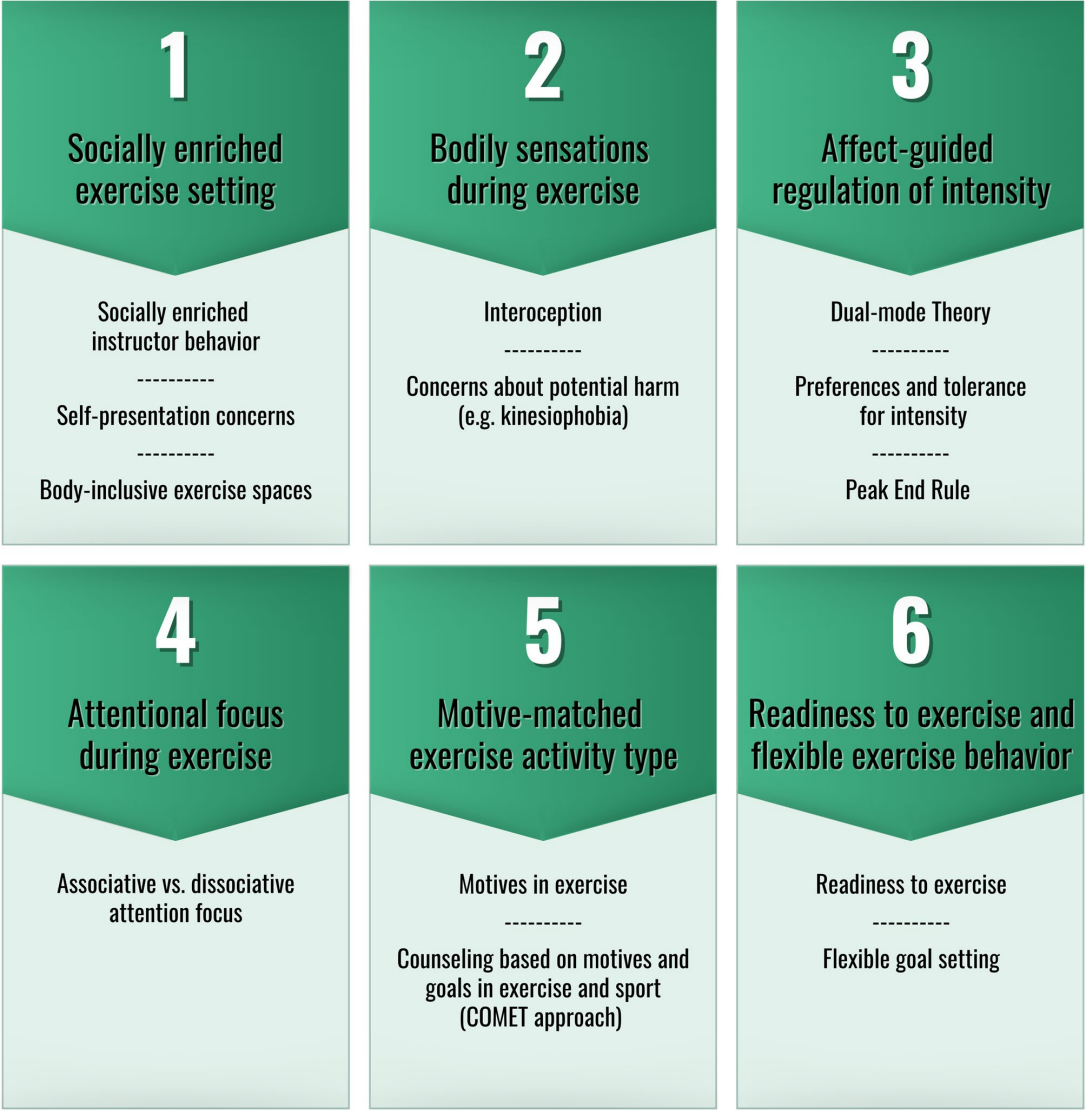
The six key elements of the FEEL exercise program and their central conceptual reference points. The supporting literature for each reference point can be found in the description of the respective key element below

To address this gap, we developed the FEEL exercise program for inactive individuals, which offers a more comprehensive approach to enhancing PAAR and affective exercise experiences. The FEEL program is structured around six key elements (see Fig. 1), informed by the aforementioned research and recent systematic reviews on factors and techniques influencing affective responses to PA [25–27].

### Key element 1: Socially enriched exercise setting

A group exercise setting and the norms established within it can significantly influence participants' affective responses [28]. Especially in a society where exercise is increasingly framed as a moral obligation, physical inactivity and low fitness levels are often linked to personal irresponsibility or may lead to diminished self-worth [29]. For instance, group exercise norms (e.g., appearance-focused language) may provoke feelings of embarrassment or discomfort driven by concerns about social perception and self-presentation [30, 31]. To mitigate these negative effects, it is essential to intentionally design exercise spaces that shield individuals from adverse (self-) evaluations [32] and actively promote group cohesion [33]. Moreover, the sometimes negative attitudes of instructors toward inactive individuals can be adjusted through intense empathy-evoking exercises [34] to create a more welcoming and socially enriched instructor behavior [35–38].

### Key element 2: Bodily sensations during exercise

Negative associations, such as fears of physical harm, can hinder regular engagement in exercise [39]. This is particularly relevant for individuals resuming regular exercise, where the likelihood of experiencing uncomfortable bodily sensations during the first weeks is high (e.g., sore muscles or fatigue) [40–42]. In these situations, inactive individuals tend to interpret interoceptive signals in an overly negative manner [43–45]. This leads to heightened perceived physical discomfort for future exercise. These (habitual) outcome expectations can significantly influence affective responses during further practice [44, 46]. In chronic pain research, individuals with kinesiophobia or fear avoidance beliefs benefit from education about (neuro-) physiological processes [47] and mindfulness practices [48], e.g., instructed body scans [49]. These approaches help reduce top-down influences and provide protective effects against unwarranted concerns about potential harm [43].

### Key element 3: Affect-guided regulation of intensity

Intensity is a key factor influencing affective responses during exercise [5, 50]. According to the Dual-Mode Theory [51], individuals generally experience enhanced affect at lower intensities during exercise. However, as intensity increases, intra-individual factors such as body composition, physical fitness, PA level, and cognitive variables influence the affective response, leading to a decline in affect beyond a certain individual intensity threshold [52–54]. These differences result in varying preferences for and tolerances of exercise intensity while exercising [55]. Furthermore, recent intervention studies showed that individuals experience more positive affective responses during exercise when they regulate intensity based on affect rather than prescribed physiological parameters or perceived exertion [21, 56]. Additionally, the findings indicate that affect-guided exercise protocols lead to more positive affective memories of the activity. Besides accounting for intra-individual preferences, decreasing exercise intensity at the end of a session has been associated with a more positive remembered affect and greater anticipated positive affect for future sessions (Peak-End rule; [10, 57]). These findings underscore the importance of encouraging inactive individuals to focus on their affective states during exercise rather than solely relying on standard physiological metrics.

### Key element 4: Attentional focus during exercise

Besides exercise intensity, there are indications that attentional focus might also influence affect during exercise too [25, 58]. Dissociative attention strategies, such as listening to music [22, 59], having social interactions [27], or exercising in a green environment [23], may be particularly effective for enhancing affective responses at low to moderate intensities, as they direct attention away from bodily sensations. However, their effectiveness diminishes at higher intensities, where physiological sensations dominate [60, 61]. In contrast, associative strategies, which involve focusing on bodily sensations or actively focusing or shared affective movement with others [22, 62], may help maintain stable affect during higher-intensity exercise [63, 64]. Although evidence is less conclusive compared to exercise intensity, it remains important for inactive people to use an attentional strategy rather than no strategy at all [63].

### Key element 5: Motive-matched activity type

A match between an individual’s motives to exercise (e.g., social contact) and an activity’s incentives (e.g., having the opportunity to socialize with others while doing partner exercises) influence affective responses and exercise maintenance [65–67]. In adulthood, motives for exercise vary greatly interindividual [68, 69]. It is assumed that a person is motivated not by one sole motive but by several working simultaneously. Everyone, therefore, possesses a unique motive profile that reflects what is important to them and what is not. For inactive individuals, however, independently identifying a suitable activity is often challenging. Recent research highlights the benefits of promoting motivational competence in exercise and sport by providing these individuals with feedback on their motive profiles, exposing them to diverse exercises, and guiding them through structured reflections on these experiences shortly [24].

### Key element 6: Readiness to exercise and flexible exercise behavior

Considering an individual’s readiness to exercise, including physiological, cognitive, and emotional states, demonstrates benefits on the affective response during exercise [70, 71]. The perception of energy, mood, and physical discomfort regarding a planned activity significantly influences affective responses during exercise [46, 72]. Consequently, inactive individuals should be encouraged to adjust planned activities based on their readiness to exercise in everyday life [70, 73]. This adaptability is particularly important given the previously mentioned moral imperative surrounding physical inactivity, alongside the strict recommendations, e.g., outlined in the WHO guidelines for physical activity [74].

The present study aims to evaluate the efficacy of the 8-week FEEL program using a cluster-randomized controlled trial. Our primary outcome is PAAR, with the hypothesis that the intervention group (IG) will achieve higher scores both immediately after the program and at follow-up compared to an active control group (CG). Secondary outcomes include affective exercise experiences and motivational competence in exercise and sport, representing another affect-related competence alongside PAAR. Additionally, we examine the program’s impact on exercise behavior and habitual affective well-being, expecting the IG to outperform the active CG in all areas. Finally, we assess the basic aspects of the FEEL program's implementation to identify factors that might support its successful adoption in real-world settings.

The FEEL study targets young adults, addressing a gap in prevention, as existing programs often fail to appeal to this age group [75]. This is particularly problematic given their increased risk of mental health issues [76], making it essential to support inactive young adults in adopting long-term exercise.

## Methods

### Design

The FEEL study is a cluster randomized controlled trial with an IG (exercise program: FEEL) and active CG (exercise program: Functional Training) with three points of assessment (t1: pre, t2: post, t3: follow-up). We designed the study to investigate the superiority of the intervention condition to the control condition from an efficacy perspective [77]. Participants are allocated to each condition in a 1:1 ratio. The FEEL and Functional Training programs are both carried out in courses over eight consecutive weeks with one 90-min session per week in groups of approximately 12–15 people but have different thematic and methodological focuses. As the FEEL study is a cooperation project between the University of Bern, Switzerland, and the University of Tübingen, Germany, the multicenter study is conducted at these two sites from September 2024 to August 2025 in at least three study waves, with a total of 160 participants. We registered the trial on https://drks.de with ID number DRKS00035302 and received approval from the local ethics committees at the University of Tübingen, Germany (ID: A2.5.4–334.1_hb) and the University of Bern, Switzerland (ID: 2024–02-06). With the present study protocol, we adhere to the Standard Protocol Items: Recommendations for Intervention Trials (SPIRIT) guidelines [78] and aligned it with the Template for Intervention Description and Replication (TIDieR) Checklist and Guide [79].

### Study setting

The FEEL study focuses on primary and secondary prevention in a non-clinical setting without therapeutic interventions. In alignment with Germany's "Guidelines for Prevention" [80], we focus the two key preventive principles:"Reducing physical inactivity through health-enhancing PA (HEPA)"and"Preventing and reducing health risks through HEPA". The two study sites, Tübingen, Germany, and Bern, Switzerland, are urban university cities surrounded by predominantly rural areas. The FEEL and Functional Training programs take place in local municipal or university halls. Additionally, in Tübingen, they are embedded in the university sports and student health management program but are accessible to students, employees, and external participants alike.

### Participants eligibility

Inclusion criteria for participants are an age between 18 and 35 years and a good level of written and spoken German. Exclusion criteria include a new diagnosis of a mental illness in the last six months by a doctor or psychiatrist (list of diseases based on [76] and regular exercise during leisure time in the last four weeks (> 0 min; BSA-F questionnaire; [81]).

### Instructors’ eligibility

Instructors leading the courses in both the intervention and control conditions must meet relevant qualifications, such as academic studies, vocational training, or certifications in fitness and exercise, along with experience in leading group-based courses.

Instructors for the intervention condition (FEEL) are primarily project members with prior experience in the program's development and piloting phases. In contrast, instructors of the control condition (Functional Training) are recruited externally to minimize diffusion bias.

### Interventions

#### Development of the intervention group program FEEL

**Fig. 2:**
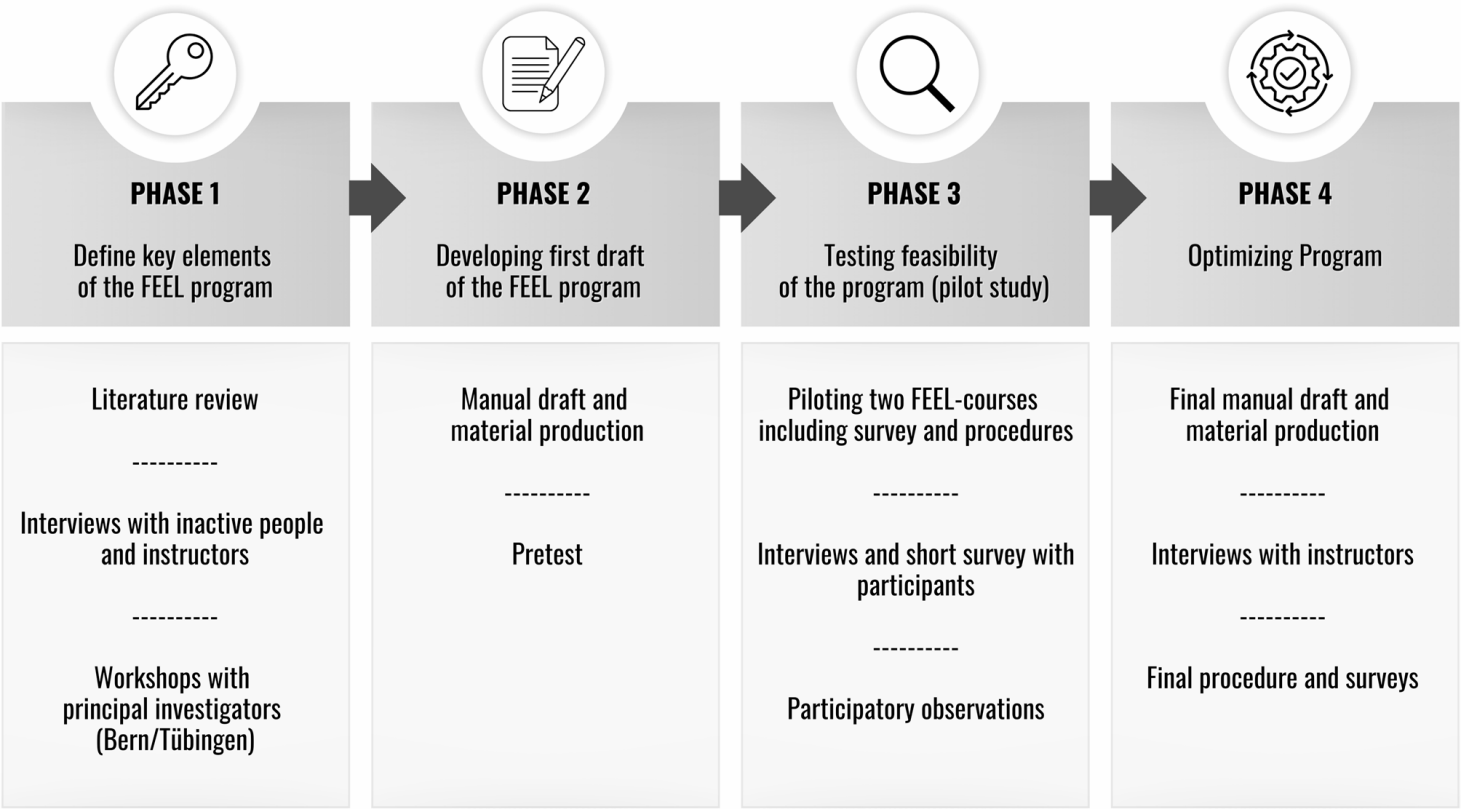
The four development phases of the FEEL program

We designed the FEEL program in four phases over 11 months (see Fig. 2). In the first two phases, we concentrated on developing the intervention [77], while in the final two phases, we assessed its feasibility [77].

In phase 1, from September 2023 to February 2024, we identified the six key elements of the FEEL program (see Fig. 1) through comprehensive literature reviews and the examination of theoretical frameworks. Additionally, we conducted several interviews with inactive individuals to understand their perspectives on exercise and with experienced instructors to gather insights on how they promote PAAR and positive affective exercise experiences in their courses. An overview with further details on the interviews can be found in the appendix (Appendix 1). Finally, we held two workshops with the principal investigators to define the FEEL program's objectives, session topics, and activities based on the six key elements.

In phase 2, from February to April 2024, we developed the first draft of the program manual and accompanying materials. We pretested each session in small groups to refine content and delivery during this time.

In phase 3, from April to July 2024, we conducted a pilot study to test the program’s feasibility, acceptance, registration process, and survey procedures. We ran the FEEL program with 10–15 participants at each site. To gain a comprehensive understanding, we took the following actions: a) we conducted focus group interviews with participants in Bern to gather qualitative feedback (see Appendix 1), b) we administered short surveys after each session with participants in Tübingen to capture immediate impressions; c) we asked instructors to document their experiences with the sessions in standardized protocols and d) we had a staff member participate in each session to document observations in a standardized protocol.

In phase 4, from July to September 2024, we optimized the program based on insights from the pilot study. Results from participants'focus group interviews, short surveys, instructor experiences, and staff member observations were synthesized. Each session was reviewed, and any necessary revisions were documented. Main revisions included enhanced time and exercise management, better alignment of session content, consistent processes, and terminology, a more structured integration of materials, and the introduction of session handouts summarizing key content for participants. Before finalizing the program manual, we conducted focus group interviews with course instructors to gather feedback on its design and usability (see Appendix 1). Additionally, we established the final procedures and surveys.

#### The final intervention group program FEEL

**Fig. 3:**
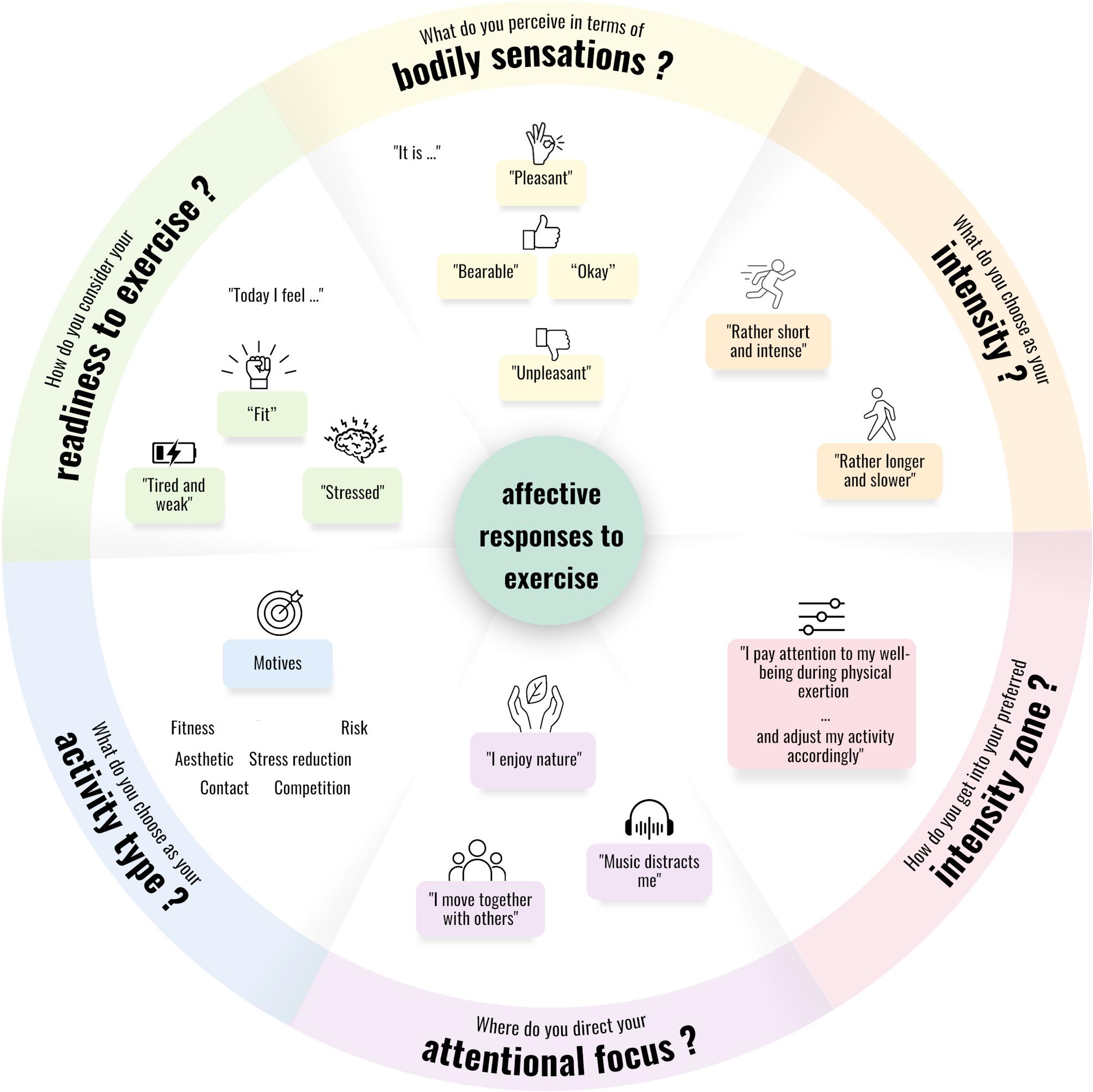
The FEEL Wheel gives participants an overview of the topics of the FEEL program

The FEEL program aims to enhance participants'PAAR while promoting positive affective exercise experiences, motivational competence, exercise volume, and habitual affective well-being. These goals are achieved through a tailored exercise program grounded in the six key elements outlined in the introduction (see Fig. 1). The FEEL program incorporates four core messages, which are presented to the participants: *“Your well-being is central”*; *“Everyone is unique, especially in how they experience exercise”*; *“Discover your own style of engaging in exercise”* and *“Celebrate your small achievements and be patient with yourself”*. Additionally, the so-called FEEL Wheel (see Fig. 3) guides participants through the program by offering an overview of the topics covered in the sessions.

The FEEL program is built upon the principles of experiential learning [82, 83], meaning that participants engage in structured exercise experiences followed by a reflection phase. Specifically, the learning cycle involves the following steps: (1) Concrete Experiences: Participants engage in specific exercises; (2) Reflective Observation: They reflect on their experiences individually or in group discussions; (3) Abstract Conceptualization: They analyze their experiences to identify underlying principles, which enables them to generalize and place their insights in a broader context; and (4) Active Experimentation: they apply acquired skills in new situations, fostering skill development and boosting self-confidence.

**Fig. 4:**
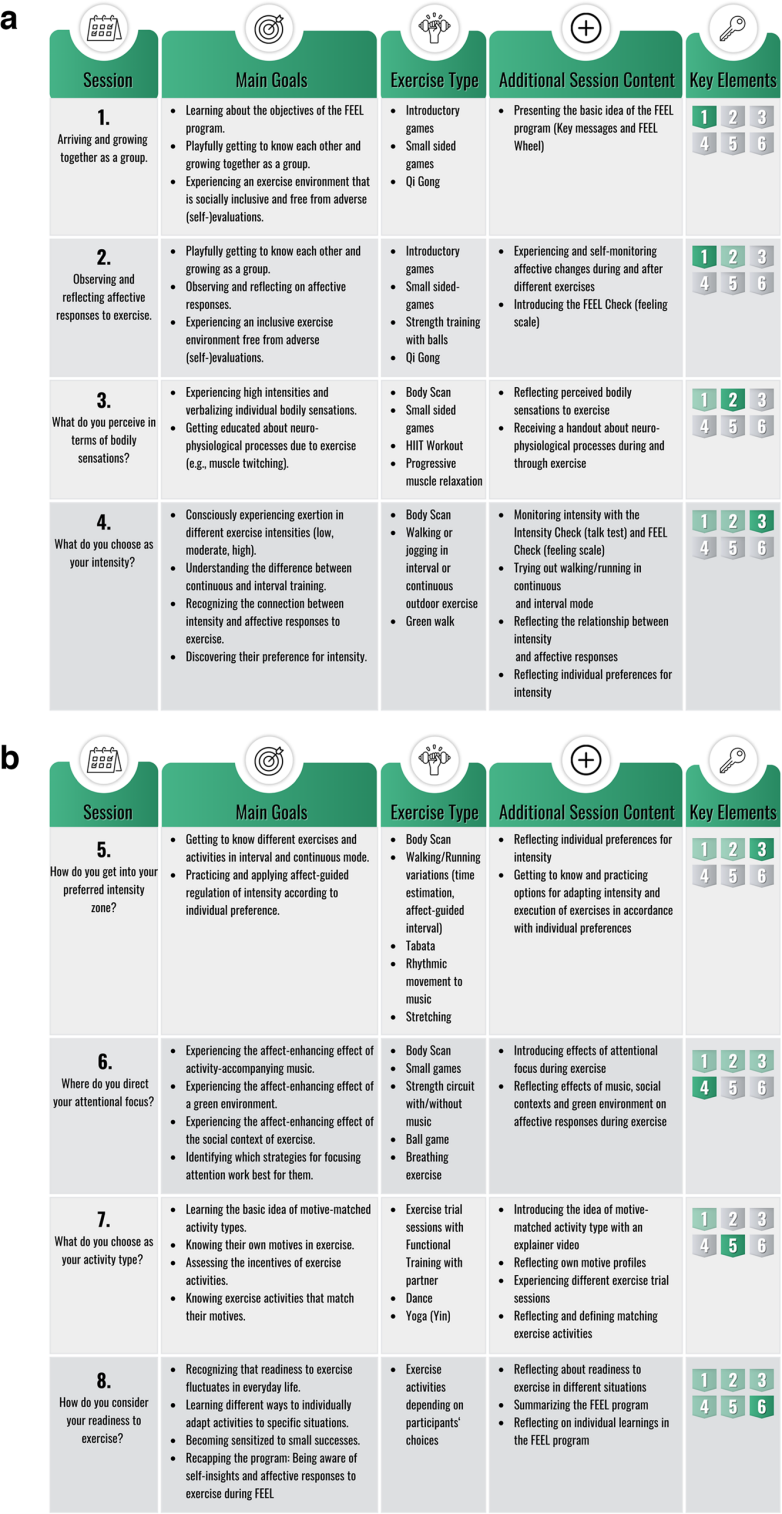
**a**, **b** give an overview of the FEEL program

Figure 4a and b give an overview of the FEEL program with its session topic, session goals, exercise type, and additional session content derived from the six key elements (see Fig. 1). Each session follows a consistent structure, ensuring a balanced combination of exercise activities and reflection aligned with the program’s objectives. The structure of the sessions includes:

- Start: Welcome participants, review the previous session, and introduce the session’s topics and activities using the FEEL Wheel.
- Ritualized Start/End: Rituals such as Body Scans, a mobilization routine, and a shared group gesture to say goodbye help to grow together as a group.
- Warm-Up: Light cardiovascular exercises or small-sided games that contribute to Concrete Experiences matching the session's goal.
- Main Part: Diverse exercise activities, including strength or endurance exercises, dance, or small-sided games, emphasize Concrete Experiences and Reflective Observation.
- Integration of Experiences: Walk-and-Talk discussions or other reflection tasks that help participants consolidate their experiences and transition into Abstract Conceptualization. These moments may occur directly after the main part or at the end of the session.
- Review: Summarize sessions’ activities, linking them to the FEEL Wheel, provide a"Message to Go"connected to the core messages of the FEEL program, and preview the next session.
- Relaxing cooldown: Each session concludes with a relaxation or a mindfulness exercise.

#### The control group program Functional Training

The active control group program is a Functional Training designed to empower participants to independently engage in Functional Training tailored to their individual fitness level while ensuring safety and effectiveness. It is based on a standardized and certified curriculum that complies with Germany's"Guidelines for Prevention"[80] under the German Prevention Law (§ 20 SGB V). To be eligible for reimbursement by health insurance companies, these programs must be certified by the Central Prevention Testing Center [80, 84]. We have adapted the Functional Training program to fit the specific requirements of this study (e.g., course sessions and duration) while ensuring alignment with the ACSM Guidelines for Exercise Testing and Prescription [85] and focusing on the objectives of health sports training [86]. See Table 1 for a one-to-one comparison with the intervention group program.

The Functional Training program employs an instructor-centered teaching approach, incorporating knowledge input, demonstration, and imitation. Each session of the Functional Training program (see for an overview Appendix 2) follows a consistent structure, which includes:

- Start: Welcome participants and introduce the session’s topics and activities.
- Knowledge input: Provide theoretical input from the instructor, focusing on Functional Training principles and techniques.
- Warm-up in four phases: a) mobilization, b) coordination exercises (“mental warm-up”), c) light cardiovascular exercises (“general warm-up”), and d) targeted exercises to prepare the body for the upcoming movement techniques practice (“warm-up in the box”).
- Movement techniques: Teach and practice key techniques from Functional Training, such as squats, push-ups, and planks.
- Fitness training: Dynamic fitness training variations such as “strength circuits” (rotational strength exercises), “As Many Rounds As Possible” (time-based strength and endurance exercise aiming for maximum rounds or reps) or “I Go, You Go” (partner-based interval training).
- Cool-down: Stretching and flexibility exercises.
- Conclusion: Wrap up with a recap of the session, preview the next session, and a farewell.

**Table 1:** Summary and comparison of the FEEL and Functional Training programs

	**Intervention group program FEEL**	**Control group program Functional Training**
Topics	1. Arriving and growing together as a group 2. Affective responses to exercise 3. Bodily sensations 4. Intensity 5. Preferred intensity zone 6. Focus of attention 7. Activity type 8. Readiness to exercise Plus: experiencing a socially enriched exercise setting	1. Functional Training basics & Warm-up 2. Breathing 3. Core 4. Stretching 5. Regeneration 6. Perceived Exertion 7. Interval training 8. Progression Plus: exercise techniques
Delivery (methodological approach)	Principles of experiential learning (structured practical experience, reflection on these experiences)	Instructor-centered teaching method (knowledge input, demonstration and imitation)
Physical environment	7 sessions indoors (municipal/university halls) 1 session outdoors	8 sessions indoors (municipal/university halls)
Social environment	Group-based	Group-based
Frequency	8 sessions (once a week) à 90 min	8 sessions (once a week) à 90 min
Intensity	Individualized according to preference (low, moderate. high), affect-guided exercise	Individualized according to fitness level (low, moderate, high)
Type/mode	• Strength exercises • Endurance exercises • Flexibility and mobilization • Dance • Small-sided games • Relaxation and mindfulness exercises	• Strength exercises • Endurance exercises • Flexibility and mobilization

#### Training of the program instructors

We provide training for instructors of both the FEEL and the Functional Training program. These trainings cover overarching aspects such as the program objectives and structure. Additionally, we review the individual course sessions by discussing their topics, main goals, exercise types, and additional content. Finally, we introduce instructors to the program manual and demonstrated selected content through practical examples. For FEEL program instructors, a supplementary workshop focuses on fostering a socially enriched exercise setting (key element 1; see Appendix 3 for further information).

After the initial training, we provide ongoing coaching throughout the program. For the FEEL program, we meet with instructors to discuss every course session in advance. For the Functional Training program, we schedule coaching sessions ahead of the third and sixth-course sessions. Regardless of the program, all instructors can reach out to one of the principal investigators at any time during the course period to ask questions or address issues as part of our troubleshooting support.

### Sample size

We conducted the sample size calculation a priori to the main study using the powerlmm package in R (Version 4.2.2). Based on prior research [14, 20, 87, 88], we assumed an effect size of *d* = 0.5 for differences in PAAR (primary outcome) between the IG and the CG over time (t1, t2, t3), with a statistical power of ≥ 80% and a two-sided significance level of α = 0.05. Additionally, we set the baseline intraclass correlation coefficient (ICC) to ICC = 0.5 and the random slope variance to 0.02. We anticipated a dropout rate of 20% at both after t1 and after t2. Accordingly, the calculation determined a minimum required sample size of N = 160 participants.

### Procedure

We recruit participants separately by study site starting several weeks in advance of the respective course periods (waves). Recruitment takes place within the respective universities, e.g., via university sports, student and occupational health management, student councils, and student organizations, at events (e.g., introductory weeks, Mental Health Days), in specific groups such as the student choir or trainees and at certain central locations (canteen, library). Participants outside the university are recruited via local companies and social media advertising. In all cases, various print (e.g., posters, flyers) and digital media (e.g., mailings, newsletters, websites, digital displays and platforms, posts/reels in social media and chat groups, or on companies'intranet) are used.

**Fig. 5:**
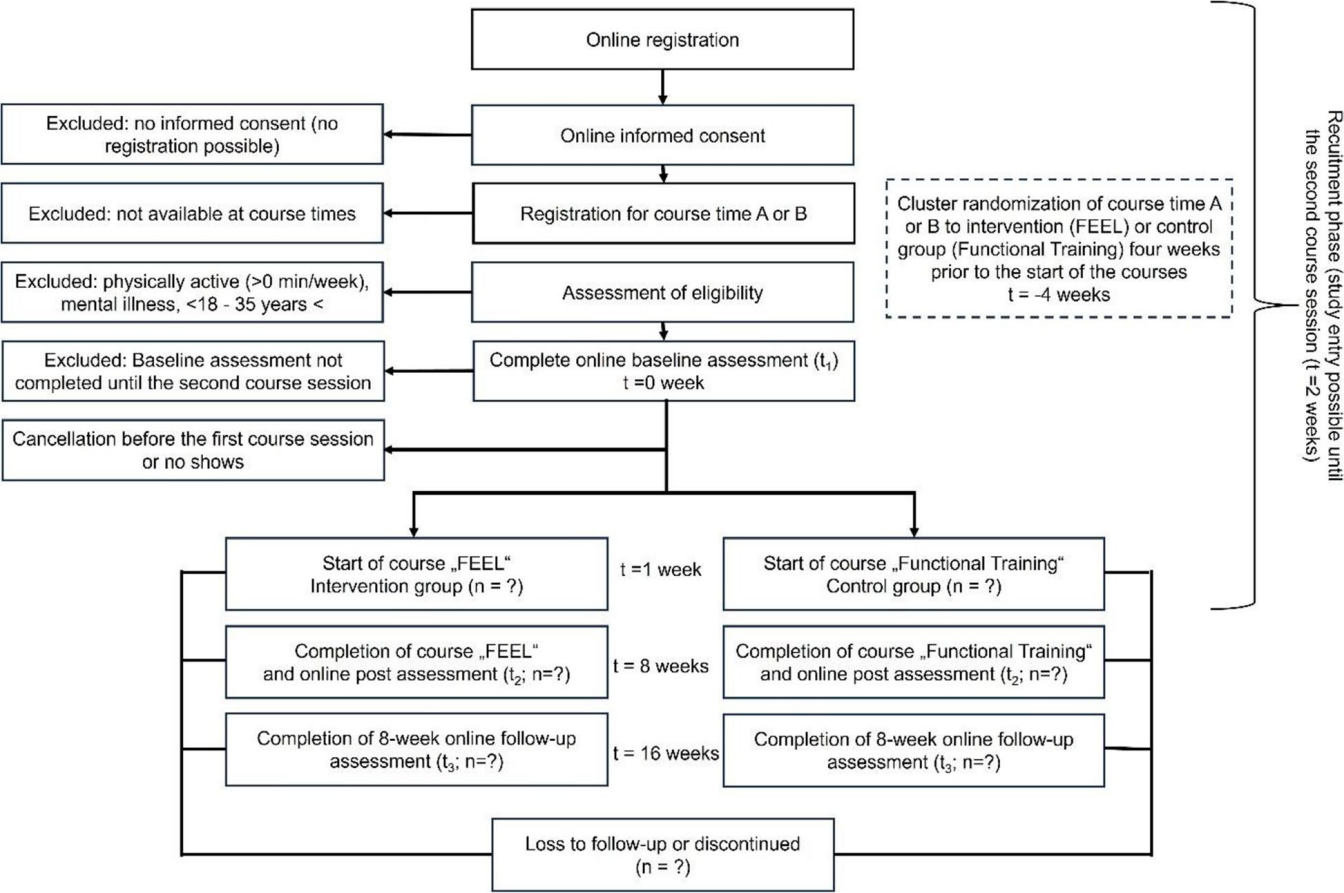
Study flow chart

The recruitment material includes a link to a website where individuals can find detailed information about the study and data protection (see Appendix 4). The website also mentions that we offer two distinct exercise programs explicitly designed for those returning to or starting exercise. These programs focus on providing exercise tailored to personal needs. After giving their informed consent, individuals can register for the exercise programs (see Fig. 5). They can choose their preferred course time (e.g., course time A or B), provide their contact details, and complete a questionnaire to check the inclusion criteria. Registration is possible until the second-course session.

During the registration phase, we continuously monitor the course enrolment and the inclusion criteria of registered participants. Individuals not meeting the inclusion criteria will be notified by email, and their registration will be canceled. Suppose there is an uneven distribution of registrations among eligible participants. In that case, a blinded project staff member will assess whether participants can switch to another course or if a certain course time should be temporarily withheld from registration.

One week before the course begins, we send the baseline questionnaire (t1) via email, requesting that participants complete it within a week. A reminder is sent three days before the completion deadline. If the questionnaire has not been completed by the deadline, we reach out personally. Within the first week after the course begins, participants who attended the first session but have not completed the questionnaire are contacted again.

One day before the course starts, participants receive a separate e-mail with directions to the course location and the contact details of the course instructor. Additionally, if an individual was absent without notice from the first session, we personally reach out in a friendly manner to inquire about their participation. A blinded staff member is responsible for all communication with the participants. Instructors also contact participants who have missed two consecutive sessions to encourage their continued participation.

After the last course session and 8 weeks after completion, participants receive an e-mail inviting them to complete the post (t_2_) and follow-up (t_3_) questionnaires. The reminder process will correspond with the baseline survey.

Participation in the FEEL and Functional Training program is free of charge. As an incentive, three €50 vouchers are raffled per site and waved among those who complete all three surveys. Instructors are compensated according to current standard rates per course session, which includes time for preparation and training.

Participants who either formally announce their withdrawal from the exercise program or fail to attend without prior notice are classified as dropouts. We record dropout time. Participants can voluntarily provide reasons, which are categorized as follows:

- Health (e.g., injury, illness)
- Time (e.g., professional or personal commitments, unable to attend scheduled sessions)
- Peers (e.g., a colleague was not accepted into the program)
- Personal reasons (e.g., bereavement)
- Program not appealing/disappointing; lost interest
- Language barriers (e.g., instructor realized during the program that they have limited German proficiency)
- No show/no reason provided

### Randomization

Cluster randomization occurs four weeks before the course begins (see Fig. 5) for practical reasons: the FEEL and Functional Training programs require indoor spaces in municipal or university halls, which are in high demand. The courses are scheduled at fixed times during each wave, and instructors must be available at these times.

The cluster randomization is conducted separately for the two study sites. This stratification ensures that the courses within each study site are evenly distributed between IG and CG. The assignment of course time A and course time B to either the intervention or control condition is determined by a computerized random number generator (random.org) operated by a researcher not involved in the project. We apply an equal allocation ratio of 1:1, meaning there is a 50% probability that either course time A or B will be assigned to the intervention, with the other time allocated to the control condition.

Table 2 shows the planned allocation of the study groups. We expect an average group size of 12 participants. This leads to a slightly larger total sample size than the required *N* = 160 participants (see section on sample size above).

**Table 2:** Planned allocation of the study groups

	Total	Wave 1						Wave 2				Wave 3			
Course clusters	7	A1		B1		B2		A2		B3		A3		B4	
Course allocations (ratio: 1:1)	14	IG	CG	IG	CG	IG	CG	IG	CG	IG	CG	IG	CG	IG	CG
Expected n		12	12	12	12	12	12	12	12	12	12	12	12	12	12
Sum n	168	24		24		24		24		24		24		24	

A = study site A, B = study site B, *IG* intervention group, *CG* control group

### Blinding

The course participants are blinded to the specific nature of the intervention. During recruitment, the course descriptions are limited to elements common to both courses (see section on procedure above). Participants are informed that two distinct courses are being conducted to study their effects on health, well-being, and exercise behaviors. In all communication, both programs are referred to as the"FEEL"program.

The course instructors are not blinded due to the apparent differences between the two programs and the association of the FEEL instructors with the project. Similarly, the principal investigators and project staff are not blinded, except for the staff member responsible for participant communication during the registration and survey process. Additionally, the statistician is blinded.

### Data collection methods

#### Primary outcome

##### Physical activity-Related Affect Regulation (PAAR)

PAAR is assessed using a scale developed by Sudeck and Pfeifer ([13], see Table 3). Originally comprising four items, the scale primarily measured the competence to use PA to reduce negative affective states (e.g.,"If my mood is unpleasant, I am able to improve it through exercise"or"I can relieve built-up stress or inner tension with exercise"). Participants rate their agreement with each statement on a 5-point Likert scale ranging from 1 (strongly disagree) to 5 (strongly agree). To broaden the scale’s scope and include the competence to enhance positive affective states through PA, two additional items were incorporated: “I am able to feel revitalized or energized through PA” and “I can use exercise to relax.” These two items were formulated based on the German version of the subjective Vitality Scales [98]. In a preliminary study with 456 participants, the revised scale of PA-related affect regulation demonstrated strong factor loadings (see Appendix 5) and good internal consistency (α = 0.941). We will use the sum of the six items for further analyses.

**Table 3:** Assessments of the FEEL study

**Characteristics**		**Questionnaires**	**Timepoint**			
			**t0**	**t1**	**t2**	**t3**
			**-4 to -1 weeks**	**0 week**	**8 weeks**	**16 weeks**
***Screening and Study Inclusion***						
Diagnosed mental illness	List of diseases based on Federal Statistical Office health report [76]		x			
Demographics	Age, gender		x			
Volume of exercise	BSA-F [81]		x	x	x	x
***Primary outcome***						
PA-Related Affect Regulation		Subscale from the PAHCO-questionnaire [13]		x	x	x
***Secondary outcomes***						
Affective exercise experiences		AFFEXX [9]; German version [89]		x	x	x
Motivational competence		Questionnaire to assess motivational competence [90]		x	x	
Exercise behavior		Exercise activities in the last four weeks; BSA-F [81]	x	x	x	x
		Habitual leisure-time PA; EHIS-PAQ [91]		x	x	x
Affective well-being		WHO-5 Well-being Index ; German version [92]		x	x	x
***Potential confounding variables and control variables***						
Body-Mass-Index		Self-reported height and weight		x		
Education level		List of highest formal education		x		
Participants sympathy and perceived competence of instructors		Subscale from LMX-MDM [93]			x	
		Questionnaire to assess instructor’s motivational, adaptive, and organizational competencies [94]			x	
Perceived fitness		Subscale from PEPS [95]		x	x	x
Movement competence		Subscales from the PAHCO-questionnaire [13]		x	x	x
Control competence for physical training				x	x	x
PA-specific self-control				x	x	x
***Implementation outcomes***						
Satisfaction with the programs		Single-item question [66]				
Acceptability of the programs		AIM [96]			x	
Appropriateness of the programs		IAM [96]			x	
Feasibility of the programs		FIM [96]			x	
Treatment fidelity		Standardized notation sheets for each session			x	
Attendance rate and reasons for absences		Absence list and open-ended question			x	
Suggestions for improving the programs		Open-ended question			x	
Delivered contents and learning experiences in the exercise programs		based on research of Grüne [97]			x	
Experiences with (re-)entering exercise in the programs		Semi-structured interview				x

*Note. PA* = Physical activity

#### Secondary outcomes

##### Affective exercise experiences

To measure affective experiences associated with past exercise participation, we use the AFFEXX questionnaire [9], which was translated and validated in German by Brand et al. [89]. The questionnaire assesses the following main areas: (1) three core affective experiences — pleasure vs. displeasure, energy vs. tiredness, and calmness vs. tension; (2) six emotional-antecedent appraisals — Liking vs. Disliking exercise in groups, Showing off vs. Shying away, Physical Empowerment vs. Bodily Harm, Pride/Honor vs. Shame/Guilt, Competence vs. Incompetence, and Interest vs. Boredom; and (3) the motivational tendency toward attraction to or avoidance of exercise (Attraction vs. Antipathy). The instrument consists of 36 bipolar items rated on a Likert scale, with antithetically worded statements ranging from 1 (e.g., “exercise makes me feel worse”) to 7 (e.g., “exercise makes me feel better”). The German translation demonstrated good to excellent internal consistency for all subscales (α = 0.80–0.90; 89). For our analysis, we will use mean scores for all subscales.

##### Motivational competence

We assess motivational competence with a scale developed and validated by Schorno et al. [90]. Participants must rate four statements (e.g., “I know exactly what to look for in an exercise activity so that I will enjoy it.” or “I know what experiences to expect with different types of exercise activities.”) on a 5-point Likert scale ranging from 1 (does not apply at all) to 5 (applies exactly). The scale demonstrated good internal consistency in existing studies (24, 86; α = 0.86). We will use the sum of the four items for further analysis.

##### Exercise behavior

Exercise behavior is measured in two ways: First, we assess the actual exercise volume with the Physical Activity, Exercise, and Sport Questionnaire (BSA-F; ([81]). Participants must report up to three exercise or sport activities they engaged in over the past four weeks, including the frequency and duration of each activity. Using this data, the weekly volume of exercise and sport (in minutes) will be calculated. The questionnaire has demonstrated satisfactory validity, as evidenced by its correlation with aerobic fitness [81]. Secondly, we assess the habitual volume of leisure-time PA with the Physical Activity Questionnaire (EHIS-PAQ; [91]). We use seven items to assess PA during transportation and during leisure time, including sports activities, aerobic health-enhancing activities, and muscle-strengthening activities in a typical week. The EHIS-PAQ demonstrates acceptable reliability and moderate to strong validity across all domains except for moderate-to-vigorous PA [99]. For our analysis, we will compute the conservative Health-Enhancing Physical Activity (HEPA) Index, which combines data on transportation-related PA (excluding walking) and leisure-time PA to evaluate compliance with the aerobic PA guidelines [91].

##### Habitual affective well-being

To assess well-being, we use the German version of the World Health Organization-Five Well-Being Index (WHO-5; [92]). It consists of five statements relating to the past two weeks (e.g., “I have felt calm and relaxed.”). Participants must rate each statement on a scale ranging from 0 (at no time) to 5 (all of the time). The scale has an excellent internal consistency with Cronbach’s α = 0.92. For our analysis, we will use the mean score of all five items.

#### Potential confounding variables and control variables

##### Body mass index (BMI)

We assess body weight and height through self-reports and subsequently use this data to calculate the BMI.

##### Education level

To assess the formal education level, participants report their highest educational qualification based on six categories. We will code the responses according to the International Standard Classification of Education (ISCED) to ensure international comparability.

##### Participants’ sympathy and perceived competence of instructors

As a potential control variable, we assess participants'sympathy toward the instructor using a subscale of the German version of the Leader-Member Exchange Scale (LMX-MDM; [93]), which has been slightly adapted for the PA setting. This subscale consists of three items rated on a 7-point Likert scale. Additionally, we measure participants'perceptions of the instructor's motivational, adaptive, and organizational competencies using a questionnaire developed by Strauch et al. [94]. This questionnaire comprises three items for each of the three subdomains, also rated on a 7-point Likert scale.

##### Perceived fitness

We measure perceived physical fitness with a modified version of a subscale from the adjective list for assessing perceived physical state (PEPS; [95]). The subscale includes four items rated on a 6-point Likert scale.

##### Movement competence, control competence for physical training, and PA-specific self-control

We assess three additional facets of PA-related health competence using scales developed from the PAHCO questionnaire [13]. The scales comprise three items for PA-specific self-control, five items for movement competence, and six items for control competence in physical training. All items are measured on a 5-point Likert scale.

#### Implementation outcomes

##### Satisfaction, acceptability, appropriateness, and feasibility of the programs

To assess participants’ satisfaction with the exercise programs, we employed a single-item question with a 5-point Likert scale [100]. To measure the acceptability of intervention measure (AIM), intervention appropriateness measure (IAM), and the feasibility of intervention measure of the exercise programs (FIM), we use two items per subdomain of the German versions of the implementation outcome measures [96], also rated on a 5-point Likert scale. Additionally, semi-structured interviews will be conducted with participants from the IG after program completion. These interviews aim to provide deeper insights into participants’ experiences and perspectives on the FEEL program.

##### Treatment fidelity

We measure treatment fidelity with standardized notation sheets for each session of the exercise programs. Instructors document any deviations from the intended program delivery.

##### Attendance rate and reasons for absences

The number of attended sessions is documented by the instructors. Furthermore, we ask participants to retrospectively specify their reasons for their (potential) absences from the sessions using a self-developed open-ended question.

##### Delivered content and learning experiences in the exercise programs

To assess which contents participants perceived and the learning experiences they gained in the exercise programs, we developed items based on a previous study [97]. The 16 items are rated on a 5-point Likert scale.

##### Experiences with (re-)adopting and resuming exercise in the programs

Guided semi-structured interviews are conducted by researchers with participants and program dropouts. These interviews offer insights into individuals'experiences of (re)entering exercise and examine the facilitators and barriers they encounter.

#### Data management and monitoring

Participants complete the surveys online via the LimeSurvey.org platform, with data collection separated for the two study sites. Data is downloaded from the platform, and each participant's responses are linked through a unique code. Interviews with program participants and dropouts are audio-recorded and transcribed verbatim, with all personal data anonymized during transcription. Instructors maintain attendance lists and notation sheets after each course session.

All collected data, including survey responses, audio recordings, transcripts, and instructor documents, is stored on password-protected servers at the respective universities. After data collection is completed, datasets from both sites are merged using anonymized participant information.

### Statistical analysis

To test the hypothesis, we will use multilevel modeling to examine the treatment effects on the primary outcome (PAAR) and the secondary outcomes (affective exercise experiences, motivational competence, exercise behavior, habitual affective well-being). Each outcome will be modeled as a function of group (IG vs. CG), time (t1, t2, t3), and their interaction.

To account for the stratified randomization by study site, the study site will be included as a covariate in all analyses. Besides baseline BMI, education level, the instructors' sympathy, and perceived competence will also be evaluated as potential covariates, as they may influence the primary outcome [101]. Covariates with a significant influence on the primary outcome will be included in the analyses.

All analyses will adhere to a modified intention-to-treat (mITT) approach, excluding participants who never attended a single session. As a sensitivity analysis, we will separately evaluate participants with an attendance rate of at least 50%. Multiple imputation methods will be applied to address missing data due to dropout or non-contribution to primary outcome measurements.

## Discussion

The limited success of existing (social-)cognitive interventions in promoting PA has prompted researchers to focus more closely on affective processes, as these play a crucial role in both initiating and maintaining PA behavior. However, it’s not enough to simply offer individuals a pleasant exercise experience; it is equally important to empower them to engage in exercise autonomously in ways that elicit positive affective responses.

Current studies typically focus on single factors that influence affective responses to exercise, such as intensity. However, a critical need is for more holistic programs that account for the complex interplay of multiple factors shaping these responses. The present RCT protocol seeks to address this gap by testing the efficacy of the comprehensive exercise program FEEL. The primary outcome of this study is PAAR, while affective exercise experiences, motivational competence, exercise behavior, and affective well-being are assessed as secondary outcomes.

The FEEL exercise program stands out not only for its comprehensive approach but also for its emphasis on learning through experience. It is built on the didactic principle of experiential learning, which encourages participants to do more than simply attend − they actively engage by discussing and reflecting on their experiences and preferences. While experiential learning is a well-established method in educational science [83], it has received little attention in the field of exercise and health promotion.

The results from both pilot studies showed promising effects concerning the primary and secondary outcomes. However, specific challenges might arise during the current trial. First, there is a potential sampling bias due to the predominantly academic sample and the underrepresentation of men. Second, the different intervention waves will take place in different seasons, which may affect course attendance. Third, structural differences between the centers (e.g., the central location of sports halls and available recruitment opportunities) could lead to unbalanced participation rates. Despite these potential challenges, the comprehensive program design, standardized sessions, and adaptive recruitment strategies are expected to facilitate the successful trial implementation and hopefully provide valuable insights into the role of affective processes in promoting sustainable PA behavior.

## Trial status

Recruitment for participants in the exercise programs for the first study wave began in the late summer of 2024, with the first groups being randomized in the autumn of 2024. The final randomization is scheduled for the end of summer 2025, and the last assessments of the last wave will be conducted in late autumn 2025.

## Supplementary Information

Supplementary Material 1.

Supplementary Material 2.

Supplementary Material 3.

Supplementary Material 4.

Supplementary Material 5.

## Data Availability

No datasets were generated or analysed during the current study.

## Abbreviations

AFFEXX: Affective Exercise Experiences
ACSM: American College of Sports Medicine
AIM: Acceptability of Intervention Measure
ART: Affective-Reflective Theory
BFS: Bundesamt für Statistik (Federal Statistical Office)
BMI: Body Mass Index
BSA-F: Physical Activity, Exercise, and Sport Questionnaire
COMET: Counseling based on motives and goals in exercise and sport
CG: Control Group
EHIS-PAQ: European Health Interview Survey-Physical Activity Questionnaire
FEEL: Fostering affect-related competencies and positive affective exercise experiences for promoting a physically active lifestyle
FIM: Feasibility of Intervention Measure
HEPA: Health-Enhancing Physical Activity
ICC: Intraclass correlation coefficient
IAM: Intervention Appropriateness Measure
ID: Identification
IG: Intervention Group
ISCED: International Standard Classification of Education
LMX-MDM: Leader-Member Exchange Multidimensional Measure
mITT: Modified intention-to-treat
PAAR: Physical activity-Related Affect Regulation
PA: Physical activity
PEPS: Adjective List for Assessing Perceived Physical State
SPIRIT: Recommendations for Intervention Trials
FOSPO: Swiss Federal Office of Sports
TIDieR: Template for Intervention Description and Replication
t1: Baseline questionnaire
t2: Post questionnaire
t3: Follow-up questionnaires
WHO: World Health Organization
WHO-5: World Health Organization-Five Well-Being Index
