# Supplementary material for: Fostering affect-related competencies and positive affective exercise experiences for promoting a physically active lifestyle in inactive young adults: study protocol for the FEEL cluster randomized controlled trial
Source: BMC Public Health. 2025 Nov 28;26:137. doi: 10.1186/s12889-025-24374-9 (PMC12797374; doi:10.1186/s12889-025-24374-9)
Supplement: Supplementary file 1 — Supplementary Material 1. [file 12889_2025_24374_MOESM1_ESM.docx]

**Appendix 1.** Overview of the interviews conducted as part of the development and optimization

of the FEEL program

**Phase 1: Identification of the six key elements**

| **Interviews with inactive individuals** | |
| --- | --- |
| Participants | Eight inactive individuals |
| Date/period | January-May 2024 |
| Objective | Understand the perspective of inactive individuals on exercise |
| Guiding questions | - What experiences have you had with exercise? - What was the situation like in which you had this experience? - What positive and negative feelings do you associate with exercise? |

| **Interviews with instructors I (1:1 interviews)** | |
| --- | --- |
| Participants | Four instructors from the fields of exercise and dance therapy and one teacher educator |
| Date/period | January 2024 |
| Objective | Generate ideas for the development of the FEEL exercise program |
| Guiding questions | - What specifically would you do in an exercise program to promote positive experiences with physical activity? - How would you enable the participants to regulate their exercise independently in such a way that it has a positive effect on their well-being? |

| **Interview with instructors II (focus group)** | |
| --- | --- |
| Participants | Five experienced instructors from the fields of dance, outdoor sports, fitness, rehabilitation |
| Date/period | February 2024 |
| Objective | Generate ideas for the development of the FEEL exercise program |
| Guiding questions | - What specifically would you do in an exercise program to promote positive experiences with physical activity? - How would you enable the participants to regulate their exercise independently in such a way that it has a positive effect on their well-being? |

**Phase 3: Testing feasibility of the FEEL program**

| **Interview with participants of the FEEL pilot program (focus group)** | |
| --- | --- |
| Participants | Five participants of the FEEL pilot program |
| Date/period | June 2024 |
| Objective | Optimization of the FEEL exercise program |
| Guiding questions | - What experiences did participants have in the FEEL exercise program? - Which elements of the FEEL program were helpful? Why? - Which elements of the FEEL program should be improved? Why? |

**Phase 4: Optimization of the FEEL program**

| **Interview with experienced instructors and teacher educators (focus group)** | |
| --- | --- |
| Participants | Three experienced instructors and teacher educators |
| Date/period | June 2024 |
| Objective | Assess the practicality of the manual using the example of session 5 |
| Guiding questions | - How do they evaluate the structure of the manual? - Which information in the manual is particularly important to them? - Do they have any suggestions for improvement of the manual? |
