# Supplementary material for: Fostering affect-related competencies and positive affective exercise experiences for promoting a physically active lifestyle in inactive young adults: study protocol for the FEEL cluster randomized controlled trial
Source: BMC Public Health. 2025 Nov 28;26:137. doi: 10.1186/s12889-025-24374-9 (PMC12797374; doi:10.1186/s12889-025-24374-9)
Supplement: Supplementary file 2 — Supplementary Material 2. [file 12889_2025_24374_MOESM2_ESM.docx]

**Appendix 2.** Overview of the content and activities of the Functional Training program

| **Session 1** | **Session 2** | **Session 3** | **Session 4** | **Session 5** | **Session 6** | **Session 7** | **Session 8** |
| --- | --- | --- | --- | --- | --- | --- | --- |
| Introduction to Functional Fitness & Warm-Up: An optimal warm-up | Power of Breath: The correct breathing techinque | Core Catalyst: A strong base | Stretch and Flow: Optimal mobility | Recharge and Renew: Good regeneration | Mastering exertion: The feeling of exertion | Pushing Limits: Interval training | The Finishing Touch: Plannig the Trainings |
| Materials: - | Materials: Resistance band | Materials: Medicine ball | Materials:  Tennis balls | Materials: - | Materials: Resistance band | Materials:  Tennis balls | Materials: Resistance band |
| **Goals** | | | | | | | |
| 1. Participants get to know the goals of the program 2. Participants learn about the relevance of functional training for everyday life 3. Participants learn about the function and effect of warm-ups 4. Participants get to know (new) movement techniques/execution | 1. Participants learn the basic principles of internal and external breathing 2. Participants learn about the relevance of breathing in sport 3. Participants learn to breathe correctly during exercise 4. Participants learn (new) movement techniques/execution | 1. Participants learn about the relevance of a strong core 2. Participants learn (new) movement techniques/execution | 1. Participants learn about the relevance of mobility 2. Participants learn the difference between mobilization and stretching 3. Participants learn (new) movement techniques/execution | 1. Participants learn about the relevance of regeneration 2. Participants learn the difference between active and passive regeneration 3. Participants learn (new) movement techniques/execution | 1. Participants learn to assess their subjective exertion 2. Participants learn to move within a certain exertion range | 1. Participants learn about the structure of interval training 2. Participants learn about the basic effects of interval training | 1. Participants learn about different ways to organize their training in the long term 2. Participants get a final overview of the entire program |
| **Knowledge transfer – Brain Boost** | | | | | | | |
| Functional Training  Warm up   - Goals - Effects | Breathing:   - Internal and external respiration - Relevance | Core:   - Stabilizers - Effects | Mobility:   - Stretching vs. mobilization - Static/dynamic stretching - Effect | Regeneration: Supercompensation   - Active and (passive) regeneration measures | Perception of exertion:   - Stress - strain - Influencing factors - CR 10 Scale | Interval training (endurance):   - Effects - Methods (load parameters) | Training design:   - Structure of training session - Design of main part - Progression |
| **Warm-up – Heat Up Zone** | | | | | | | |
| Mobilization ritual | Mobilization ritual | Mobilization ritual | Mobilization ritual | Mobilization ritual | Mobilization ritual | Mobilization ritual | Mobilization ritual |
| Pass on signal | Tandem stands | Circle of eight | Tennis ball coordination | Command go | Color game | Tennis ball coordination | Mirror |
| Running variations in the box | Jogging in a rectangle | Star run | Shadow run | Line run | Running variations in the box | Zipper | figure-eight course |
| **Exercise techniques – Movement Mastery** | | | | | | | |
|  | Repetition | Repetition | Repetition | Repetition | Repetition |  | Repetition |
| Squat  Push-Up | Rowing  Good Mornings | Plank/Sideplank | Lunge  Core exercises | Crawling  Jumping |  |  |  |
| **Fitnesstraining – Power Play** | | | | | | | |
| Dice game | AMRAPS partly with Resistance bands | Circuit with load for time, partly with a medicine ball | I go you go | Circle of stars | AMRAPS partly with Resistance bands(rep. L. 2) | Tabata | Circuit Training |
| **Regeneration/completion – Recovery Retreat** | | | | | | | |
| Stretching ritual | Stretching ritual | Stretching ritual | Stretching ritual | Stretching ritual | Stretching ritual | Run out  Tennis ball massage  Stretching ritual | Stretching ritual |
