# Supplementary material for: Fostering affect-related competencies and positive affective exercise experiences for promoting a physically active lifestyle in inactive young adults: study protocol for the FEEL cluster randomized controlled trial
Source: BMC Public Health. 2025 Nov 28;26:137. doi: 10.1186/s12889-025-24374-9 (PMC12797374; doi:10.1186/s12889-025-24374-9)
Supplement: Supplementary file 3 — Supplementary Material 3. [file 12889_2025_24374_MOESM3_ESM.docx]

**Appendix 3**. FEEL instructor workshop focusing on socially enriched exercise environment

| **Main goals** | **Workshop content** | **time** |
| --- | --- | --- |
| 1. Instructors can empathize with the feelings of inactive people in exercise environments | - “Challenging” exercise experience: Instructors explore two new exercise activities on their own before the workshop. These activities should be unfamiliar and challenging, for example, by preventing them from relying on prior experience. - Reflective report: Instructor reflect on experiences and feelings in a journal. - Exchange: All instructors come together to discuss their experiences and feelings. | 180 minutes |
|  | - Case studies: Instructors watch and discuss videos featuring two inactive individuals describing their affective experiences with exercise. | 10 minutes |
| 1. Instructors understand how affective exercise experiences influence exercise behavior | - Theoretical framework: Presentation of the Affective-Reflective Theory (8) and the Affective Exercise Experiences (AFFEXX) framework (9). Instructors summarize the key points of the two models. | 20 minutes |
| 1. Instructors learn strategies to make inactive people feel welcome and comfortable in exercise environment and to help them grow together as a group. | - Best- and Worst Case: Instructors create and discuss a best-case and a worst-case scenario for the exercise program. The guiding questions are: What does an exercise environment look like where participants feel (un)welcome and (un)comfortable? - Developing strategies: Presentation of the framework for Body-inclusive physical activity spaces (32). Instructors develop and discuss concrete strategies for fostering socially enriched exercise environment. | 60 minutes |
