# Supplementary material for: Fostering affect-related competencies and positive affective exercise experiences for promoting a physically active lifestyle in inactive young adults: study protocol for the FEEL cluster randomized controlled trial
Source: BMC Public Health. 2025 Nov 28;26:137. doi: 10.1186/s12889-025-24374-9 (PMC12797374; doi:10.1186/s12889-025-24374-9)
Supplement: Supplementary file 4 — Supplementary Material 4. [file 12889_2025_24374_MOESM4_ESM.docx]

**Appendix 4.** Informed Consent Materials

**Who can participate in the study?**

The study is open to individuals between 18 and 35 years old who would like to become physically active. You should not currently engage in regular sports activities. Additionally, good proficiency in German, both written and spoken, is required.

**How does the study work?**

In the FEEL study, we will conduct two 8-week exercise programs (one session per week lasting 75–90 minutes). A random selection process will determine which program you participate in. The exercise programs will take place in small groups and will be guided by exercise specialists. They are designed for beginners or those returning to exercise and do not require prior sports experience or a high fitness level. The sessions will be held in a gymnasium in the city of [blended for review] or outdoors in nature.

As part of the project, we will collect data at multiple time points. During registration for the exercise programs, we will collect basic personal information and details about your physical activity to determine if you meet the study's eligibility criteria. At the start of the exercise programs, immediately after their completion, and again two months later, we will conduct an online survey to ask about your exercise experiences, health, and overall well-being. Completing these surveys will take approximately 20 minutes each time.

**Is participation voluntary, and can I withdraw at any time?**

Yes! Participation in the study is completely voluntary. There will be no disadvantages if you choose not to participate. Additionally, you may withdraw from the program at any time without providing a reason.

**Are there any risks or burdens for participants?**

Participating in this study involves only minimal risks. The study does not include invasive procedures such as blood draws. However, any physical activity carries a risk of injury. Exercise specialists will supervise the sessions to minimize this risk as much as possible. If you are ill (e.g., with the flu or similar), participation is discouraged. Participants are responsible for their own insurance coverage.

**How is data protection handled?**

As part of this study, your personal data will be processed in a pseudonymized form. This means that all participants will be assigned a unique participant code at the beginning. A list linking your personal information (name, phone number, email address) to your code will be stored in a password-protected local folder on a server at the Institute of Sports Science at the University of [blended for review]. This is necessary to compile all collected information about you. Only the project team has access to this list. The list will be deleted five months after the last data collection. From that point on, personal identification of the data will no longer be possible, and the data will be fully anonymized.

The anonymized data will be stored for at least 10 years and may be used for further research purposes. As part of a research collaboration with the University of Tübingen, anonymized data will be shared and compiled into a joint dataset for analysis. The study’s research results will be published in anonymous form in academic journals or scientific databases. Your identity will not be disclosed in any published research findings.

You can request the deletion of your data up to five months after the second data collection. To do so, please contact our project staff member, xy, with your participant code at [blended for review]**@uni.com** or call [blended for review].
