# Supplementary material for: Fostering affect-related competencies and positive affective exercise experiences for promoting a physically active lifestyle in inactive young adults: study protocol for the FEEL cluster randomized controlled trial
Source: BMC Public Health. 2025 Nov 28;26:137. doi: 10.1186/s12889-025-24374-9 (PMC12797374; doi:10.1186/s12889-025-24374-9)
Supplement: Supplementary file 5 — Supplementary Material 5. [file 12889_2025_24374_MOESM5_ESM.docx]

**Appendix 5.** Adapted scale for measuring Physical activity-related affect regulation (PAAR)

| **Abbre-viation** | **Item wording German** | **Item wording English**^1^ |
| --- | --- | --- |
| PAAR1* | Mir gelingt es gut, meine gedrückte Stimmung durch Bewegung zu verbessern | If my mood is unpleasant I am able to improve it through exercise. |
| PAAR2* | Ich bin in der Lage, durch körperliche Aktivität meine Stimmung zu regulieren. | I am able to regulate my mood through physical activity. |
| PAAR3* | Wenn es mir schlecht geht, kann ich mich durch körperliche Aktivität gut ablenken. | When I am feeling down, I can distract myself with physical activity. |
| PAAR4* | Ich kann aufgestauten Stress und innere Anspannung durch Bewegung gut wieder abbauen. | I can relieve built-up stress or inner tension with exercise |
| PAAR5^#^ | Ich kann mich durch Bewegung gut entspannen. | I can use exercise to relax. |
| PAAR6^#^ | Ich bin in der Lage, mich durch körperliche Aktivität vital und lebendig zu fühlen. | I am able to feel revitalized or energized through physical activity. |
| *Note.* Participants must rate each statement on 5-point Likert scale ranging from 1 (strongly disagree) to 5 (strongly agree); *** = original items developed and validated by Sudeck & Pfeifer (1); ^#^ = newly formulated items; ^1^ Forward-backward translation following established guidelines by Beaton et al. (2). | | |

| **Abbre-viation** | **M** | **SD** | **Cronbach**  **α** | **Factor loadings CFA** | **r_it_** |
| --- | --- | --- | --- | --- | --- |
| PAAR1 | 2.68 | 1.18 | .94 | 0.85 | .83 |
| PAAR2 | 2.38 | 1.35 |  | 0.86 | .83 |
| PAAR3 | 2.35 | 1.26 |  | 0.90 | .87 |
| PAAR4 | 2.45 | 1.25 |  | 0.88 | .84 |
| PAAR5 | 2.95 | 1.12 |  | 0.78 | .74 |
| PAAR6 | 2.47 | 1.20 |  | 0.85 | .82 |
| *Note.* Sample: N = 456 students. 74% female. 23% male. 3% divers. M_age_ = 23.9 years; CFA = confirmatory factor analysis; r_it_ = item-test correlation | | | | | |

Literature Cited

1. Sudeck G, Pfeifer K. Physical activity-related health competence as an integrative objective in exercise therapy and health sports – conception and validation of a short questionnaire. Sportwissenschaft 2016; 46(2):74–87. https://doi.org/10.1007/s12662-016-0405-4.

2. Beaton DE, Bombardier C, Guillemin F, Ferraz MB. Guidelines for the Process of Cross-Cultural Adaptation of Self-Report Measures. Spine 2000; 25(24).
